# Development and validation of an immune-related gene signature for predicting the radiosensitivity of lower-grade gliomas

**DOI:** 10.1038/s41598-022-10601-5

**Published:** 2022-04-23

**Authors:** Derui Yan, Qi Zhao, Zixuan Du, Huijun Li, Ruirui Geng, Wei Yang, Xinyan Zhang, Jianping Cao, Nengjun Yi, Juying Zhou, Zaixiang Tang

**Affiliations:** 1 Department of Biostatistics, School of Public Health, Medical College of Soochow University, Suzhou, 215123 Jiangsu China; 2 Jiangsu Key Laboratory of Preventive and Translational Medicine for Geriatric Diseases, Medical College of Soochow University, Suzhou, China; 3 Department of Radiation Oncology, The First Affiliated Hospital of Soochow University, Suzhou, 215000 Jiangsu China; 4 School of Radiation Medicine and Protection and Collaborative Innovation Center of Radiation Medicine of Jiangsu Higher Education Institutions, Soochow University, Suzhou, 215006 China; 5 Department of Biostatistics, Kennesaw State University, Kennesaw, GA 30144 USA; 6 Department of Biostatistics, University of Alabama at Birmingham, Birmingham, AL 35294 USA

**Keywords:** Cancer, Computational biology and bioinformatics, Immunology, Biomarkers, Neurology, Oncology

## Abstract

Radiotherapy is an important treatment modality for lower-grade gliomas (LGGs) patients. This analysis was conducted to develop an immune-related radiosensitivity gene signature to predict the survival of LGGs patients who received radiotherapy. The clinical and RNA sequencing data of LGGs were obtained from The Cancer Genome Atlas (TCGA) and the Chinese Glioma Genome Atlas (CGGA). Lasso regression analyses were used to construct a 21-gene signature to identify the LGGs patients who could benefit from radiotherapy. Based on this radiosensitivity signature, patients were classified into a radiosensitive (RS) group and a radioresistant (RR) group. According to the Kaplan–Meier analysis results of the TCGA dataset and the two CGGA validation datasets, the RS group had a higher overall survival rate than that of the RR group. This gene signature was RT-specific and an independent prognostic indicator. The nomogram model performed well in predicting 3-, and 5-year survival of LGGs patients after radiotherapy by this gene signature and other clinical factors (age, sex, grade, IDH mutations, 1p/19q codeletion). In summary, this signature is a powerful supplement to the prognostic factors of LGGs patients with radiotherapy and may provide an opportunity to incorporate individual tumor biology into clinical decision making in radiation oncology.

## Introduction

Radiotherapy plays a vital role in cancer treatment. Currently, more than 60% of cancer patients receive radiotherapy. Radiotherapy can significantly prolong the survival of patients and improve the local control rate of tumors; it can also be used as palliative treatment^1^. Diffuse lower-grade gliomas (LGGs) (WHO grades II/III) is a common invasive brain tumor in adults that mainly includes astrocytoma, oligodendroglioma, and oligoastrocytoma^2,3^. LGGs is highly aggressive, and it is impossible to perform complete neurosurgical resection. The presence of residual tumor can lead to recurrence and malignant progression; thus, LGGs will eventually develop into a higher-grade glioma with higher mortality^4^. The main treatment methods for LGGs include surgery, observation, chemotherapy, and radiotherapy. Radiotherapy as an adjuvant therapy is very important for LGGs treatment. Studies have shown that radiotherapy can significantly improve the overall survival (OS) and progression-free survival (PFS) of LGGs patients^5–7^. Although radiotherapy has important clinical significance, the absolute benefit of radiotherapy is related to individual characteristics, and not all patients benefit from radiotherapy^8^.

Radiotherapy directly kills cancer cells and can affect the tumor microenvironment. However, the effects of radiotherapy will be affected and regulated by the tumor microenvironment. The tumor microenvironment is known as the “game changer” in cancer radiotherapy^9^. Ionizing radiation increases tumor antigen presentation by activating the NF-κB/IFN-β/MHC I signaling axis, thereby increasing the lethality of cytotoxic T lymphocytes to tumor cells. Ionizing radiation can also affect the activation of antigen-presenting cells and natural killer cells, the release of danger signals, and the expression levels of PD-1 and PD-L1^10^. Previous research has shown that low-dose radiation can activate the immune response; this condition is called “in situ” vaccination^11^. High-dose radiation therapy can suppress the immune system and induce resistance. The complex interaction between tumor cells and the tumor microenvironment greatly influences tumor sensitivity to ionizing radiation. Exploring the impact of radiotherapy on the tumor microenvironment, rather than on isolated tumor cells, is currently one of the key goals in radiobiology^12^. Combining radiation with immunotherapy is a focus of current and future radiotherapy studies at the National Cancer Institute (NCI) to greatly improve the effectiveness of radiotherapy^8^.

Gene expression profiling has been used in many types of cancer to develop prognostic and/or predictive biomarkers, allowing for the identification of patients who can benefit or suffer limited treatment-related harm. Recently, several prognostic models constructed based on immune-related genes (IRGs) have been used to stratify risk and predict clinical outcomes in several cancer types, including breast cancer^13^, epithelial ovarian cancer^14^, hepatocellular carcinoma^15^, and lung adenocarcinoma^16^. However, most studies mainly focus on the OS of cancer patients, and few studies have explored the benefits of specific treatments^17^. There are also a limited number of studies systematically evaluating IRGs and their association with radiosensitivity in LGGs patients.

In the present study, we integrated common immune genes in The Cancer Genome Atlas (TCGA) dataset, the Chinese Glioma Genome Atlas (CGGA) dataset, and the immunology database and analysis portal (ImmPort) to construct an LGGs radiosensitivity prediction signature to identify the patients most likely to benefit from radiotherapy. The nomogram integrated signature with clinical factors was established to predict the survival rate of LGGs patients after radiotherapy. In addition, bioinformatics analysis was conducted to study the underlying mechanisms of the signature, which may lead to new ideas for future combined immune-radiotherapy.

## Materials and methods

### Collection of data from TCGA and the CGGA

**Figure 1:**
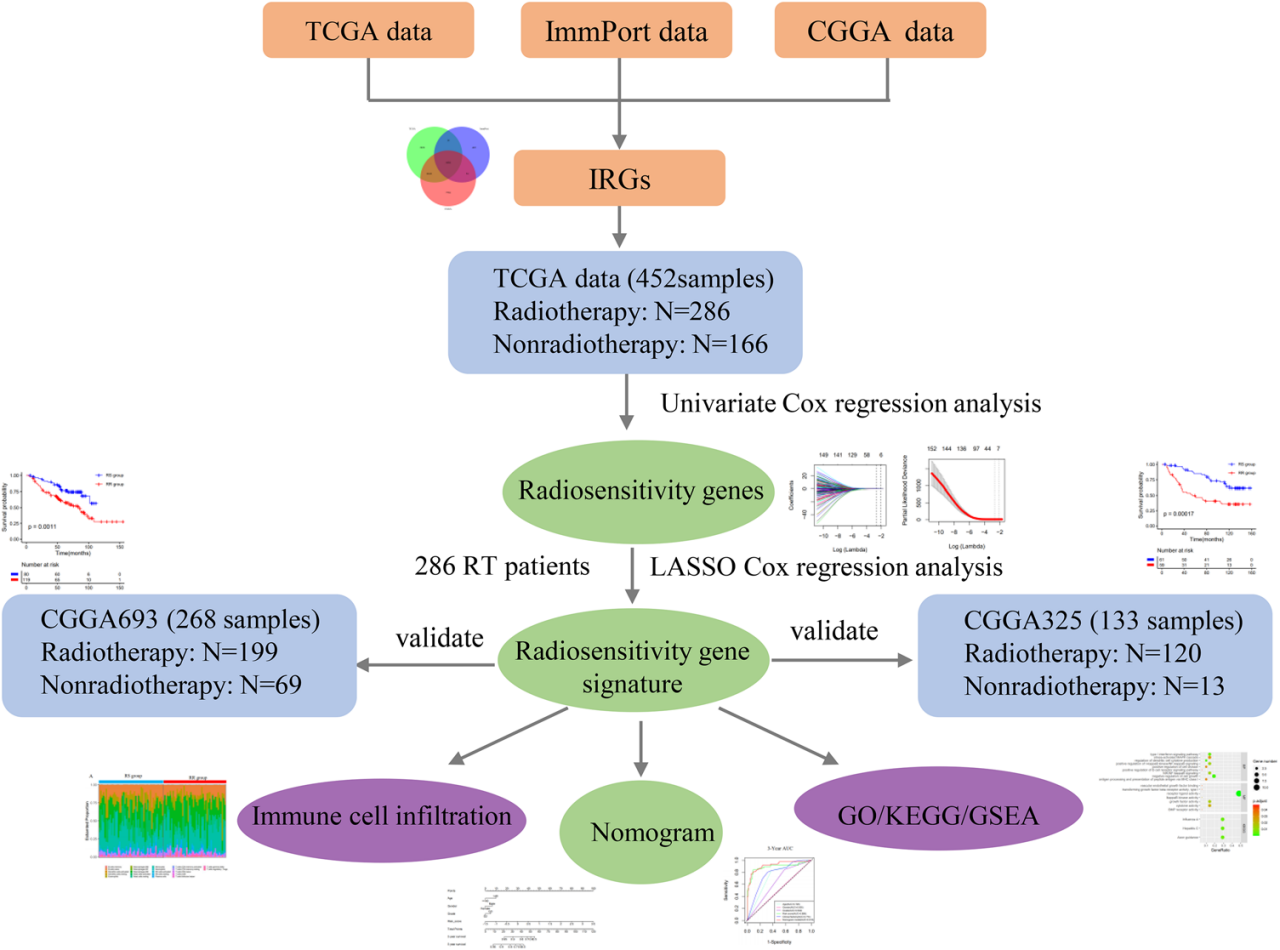
The workflow of this research.

The clinical information and mRNA sequencing expression data of LGGs patients were downloaded from TCGA using UCSC XENA data hubs (https://xenabrowser.net). Among the 452 LGGs patients in this study, the following information was included: (1) primary tumor type; (2) pathological type and grade [WHO grade II or III]; (3) complete survival time and survival status of samples with a survival time of more than 30 days; and (4) complete radiotherapy information. For mRNA data, the normalized gene expression count was measured as upper quartile normalized RSEM count estimates. Normalized gene expression data were filtered to remove genes with a maximum expression value < 10 or genes with a zero expression ratio > 50%. For external validation, the RNA-RSEM data and clinical information of two LGGs datasets were obtained from the CGGA (http://www.cgga.org.cn) database. The two CGGA datasets included 266^18,19^ and 133^20,21^ patients with LGGs. Then, the two CGGA datasets were merged, and the ComBat method was used to remove the batch effects using the R package “SVA”. The clinical information of LGGs patients is summarized in Supplementary Table S1. Figure 1 showed the workflow of this research. OS was the primary endpoint. Each dataset was individually transformed into a z-score to remove platform differences. Ethics approval statement is not needed because the samples were obtained from the public databases. All research was performed in accordance with relevant guidelines/regulations.

For the TCGA-LGGs, CGGA693, and CGGA325 datasets, we performed an exact 1:1 match on radiosensitive (RS) and radioresistant (RR) patients in each dataset according to age (≤ 40; > 40), sex (male; female), and grade (II; III). Specifically, each RS patient was accurately matched to a patient in the RR group according to the clinical information described above. If no corresponding RR patients matched, the patient was excluded from the RS group. If multiple RR patients matched, the first RR match was selected. The matched patients were removed from the cohort, and no further matches were made. The above matching process was repeated until either of the two groups completed matching, and then the matching was considered complete.

### Development and validation of the immune radiosensitivity signature

In this study, radiosensitive patients were defined as a group of patients who had better overall survival after receiving radiotherapy^22,23^. Specifically, all radiotherapy patients were divided into a low-risk group and a high-risk group according to a gene signature. Patients in the low-risk group (RS group) obtained significantly more survival benefits than the patients in the high-risk group (RR group). However, the survival difference might not indicate radiosensitivity in single radiotherapy scenario. This signature also divided nonradiotherapy patients into two groups, and it is possible that the low-risk group might also have better overall survival than the high-risk group. In this case, both the low-risk group and the high-risk group might benefit from radiotherapy with the same effect size. Therefore, it must be determined that the survival rate of the low-risk group (RS group) was not better (equal or worse) than that of the high-risk group (NRS group) when neither group received radiotherapy^24^. The result indicated that this gene signature has a significant interaction with radiotherapy (P < 0.05), this gene signature was RT-specific^22^.

The list of IRGs used in our research was obtained from the ImmPort database (https://www.immport.org/) (Supplementary Table S2). First, the common IRGs were extracted by overlapping the TCGA dataset, the CGGA datasets, and all IRGs. Next, a univariate Cox regression model was applied to identify individual genes that had significant interactions with radiotherapy in the TCGA dataset.

All radiotherapy patients in the TCGA dataset were then used for model construction. A LASSO penalized regression model was used to construct the radiosensitivity signature. Tenfold cross-validation was used to estimate the amounts of penalty, and the minimum lambda value was used as a cutoff. The following formula was used to calculate the risk score of each LGGs patient:

$$Risk\, score=\sum_{i}^{k}{\beta }_{i}{S}_{i},$$

where k, ${\beta }_{i}$, and ${S}_{i}$ represent the number of immune genes, the LASSO coefficient, and the corresponding gene expression level, respectively. We calculated the risk score for each sample and then separated the patients into high-risk and low-risk groups based on the median value. Kaplan–Meier analysis using log-rank testing was used to compare the survival difference between the two groups. The prediction signature was validated with the CGGA datasets. The “glmnet” and “survival” packages were used to perform these analyses.

### Gene functional annotation of the immune genes

To understand the potential functions of these genes, GO function and KEGG pathway enrichment analyses for the selected genes were performed using the “clusterprofiler” R package. An adjusted *P* value < 0.05 was considered significant.

### Construction and assessment of the nomogram

Retain complete datasets of clinical information. A nomogram composed of relevant clinical factors and risk scores was constructed. Calibration curves (3-year and 5-year survival) were applied to compare the nomogram prediction rates against the observed rates. Time‐dependent ROC curves were employed to evaluate the prediction accuracy in the TCGA and CGGA samples. The nomogram, calibration curves, and ROC curves were plotted via the R packages “rms” and “timeROC”.

### Tumor-infiltrating immune cell analysis

The CIBERSORT algorithm was used to calculate the relative proportions of 22 immune cell types in all LGGs patients based on IRGs. The number of permutations was set at 1000, and samples with *P* < 0.05 were selected for analysis. Furthermore, the ESTIMATE algorithm was used to determine the immune score of each sample by the R package “ESTIMATE”. The Wilcoxon test was used to assess the differences between the high- and low-risk groups.

### Gene set enrichment analysis (GSEA)

GSEA was performed to assess the differential enrichment of biological processes between the high-risk and low-risk groups. The “limma” package was used to identify the DEGs, and an adjusted *P* value < 0.05 and |log2 fold‐change (FC)|> 1 were used as cutoffs. The c2.all.v7.2.entrez.gmt collection in the Molecular Signatures Database (MSigDB, http://software.broadinstitute.org/gsea/msigdb/index.jsp) was selected as the reference gene collection.

### Statistical analysis

Statistical analyses were conducted in R version 3.6.3. The chi-square test and the Mann–Whitney U test were used to compare the differences between different groups.

A *P* value < 0.05 was considered statistically significant.

## Results

### Identification of immune-related radiosensitivity genes and functional annotations in the TCGA-LGGs dataset

**Figure 2:**
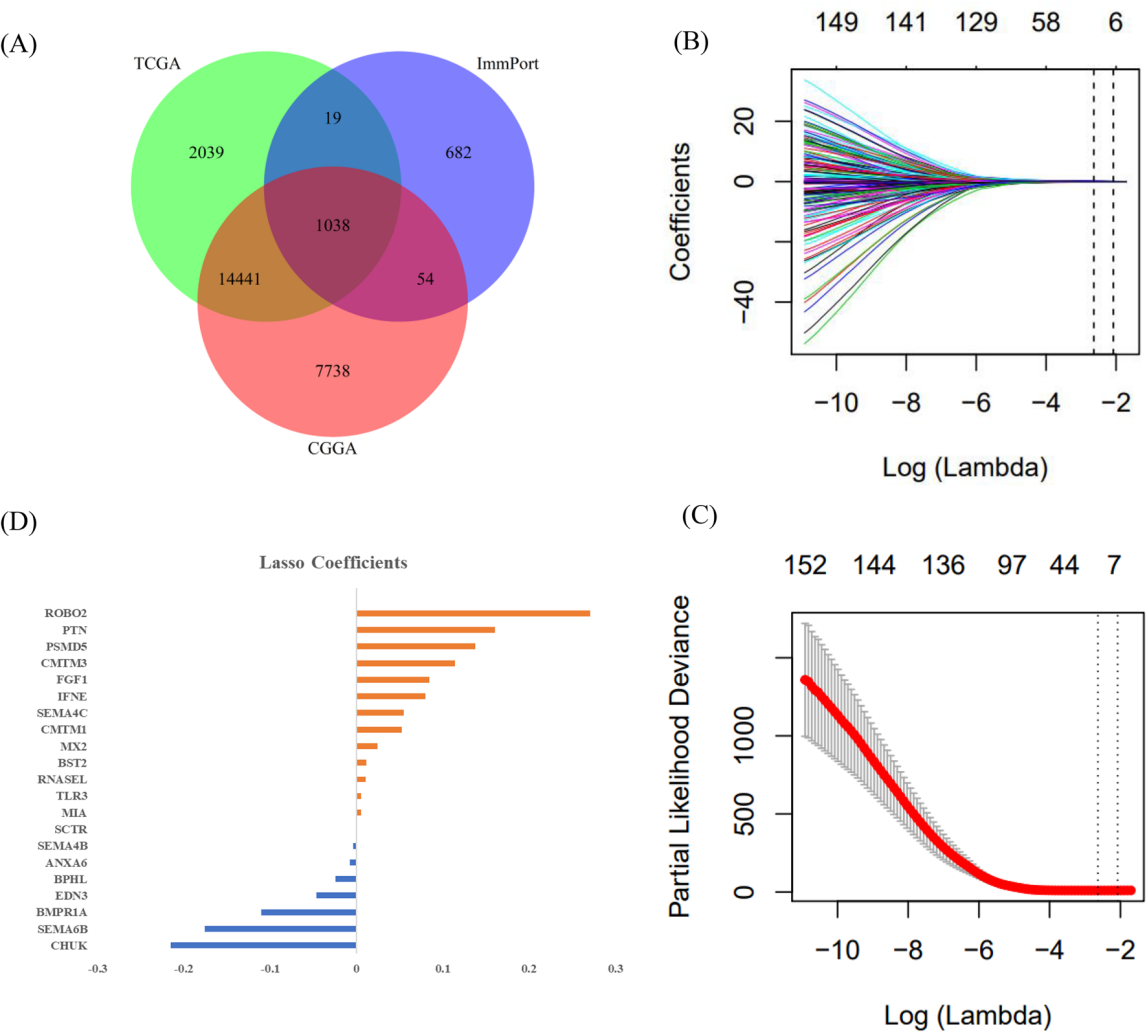
Identification of immune genes in the TCGA dataset. (**A**) The common immune genes in the TCGA-LGGs dataset, CGGA datasets, and ImmPort data. (**B**,**C**) 21 genes selected by the LASSO model. (**D**) Coefficient distribution of gene signature in the lasso model.

The RNA-seq datasets of TCGA-LGGs and CGGA were integrated with the immune gene list information in the ImmPort database, and the Venn diagram showed that 1038 IRGs were identified from the overlapping cluster (Fig. 2A). Among them, 157 genes were significantly related to the OS of radiotherapy patients (*P* < 0.05) but not to the OS of nonradiotherapy patients in the TCGA-LGGs dataset (Supplementary Table S3). LASSO Cox regression analysis further revealed 21 immune-related radiosensitivity genes (ANXA6, BMPR1A, BPHL, BST2, CHUK, CMTM1, CMTM3, EDN3, FGF1, IFNE, MIA, MX2, PSMD5, PTN, RNASEL, ROBO2, SCTR, SEMA4B, SEMA4C, SEMA6B, and TLB3) (Fig. 2B–D).

GO analysis showed that these genes were significantly enriched in negative regulation of cell growth, regulation of dendritic cell cytokine production, and antigen processing and presentation. Molecular function (MF) analysis demonstrated that these genes were enriched in receptor ligand activity, growth factor activity, and cytokine activity. In addition, KEGG analysis indicated that these genes were mainly associated with hepatitis C, influenza A, and axon guidance (Supplementary Fig. S1, Supplementary Table S4).

### Development of a radiosensitivity prediction signature with the TCGA dataset

**Figure 3:**
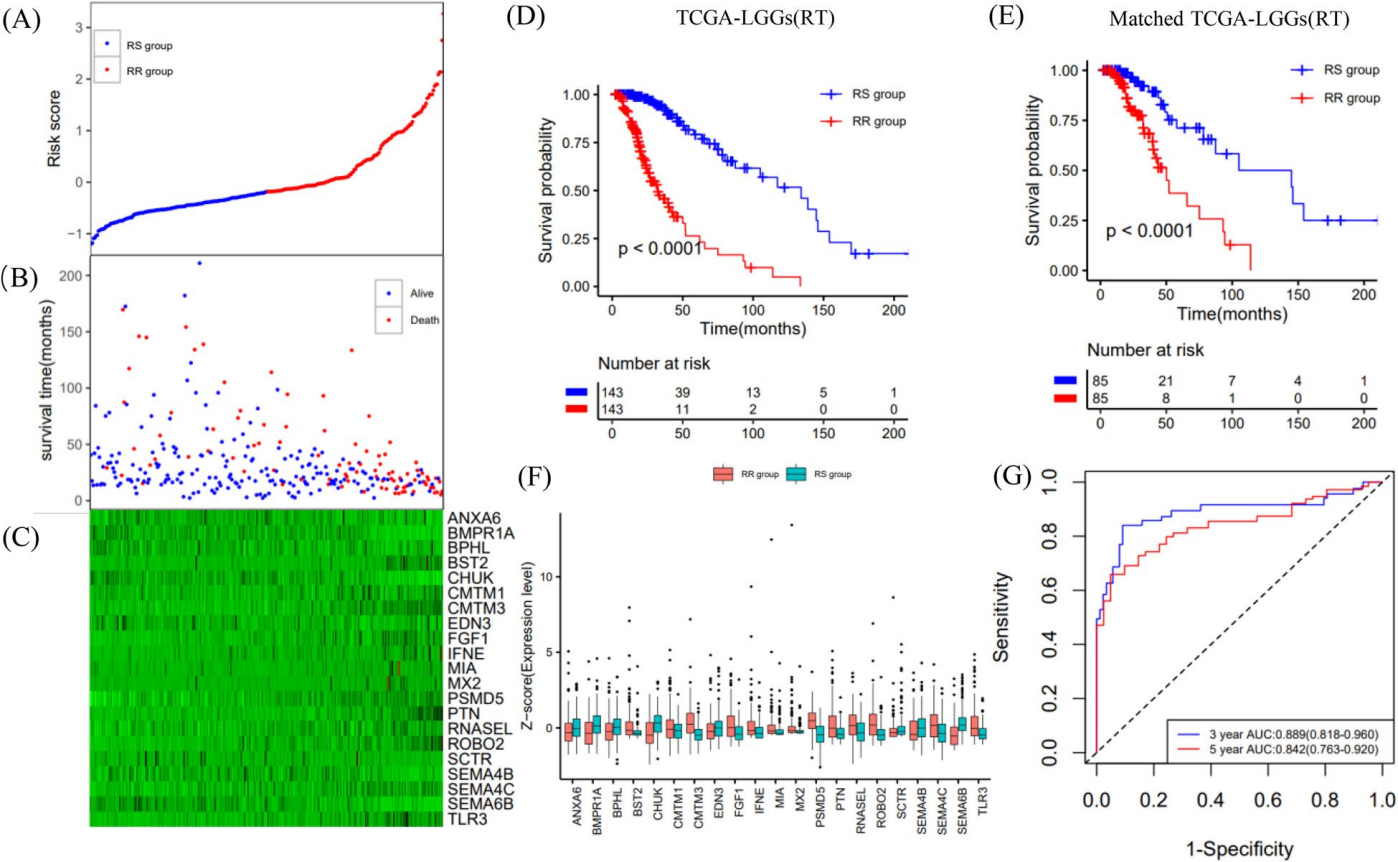
Construction of the OS prediction model based on 21 genes in the TCGA dataset. (**A**,**B**) The risk score, survival time of each sample. (**C**) Heatmap of 21 genes. (**D**,**E**) Survival curve of the RS group and RR group in radiotherapy patients in the unmatched and matched datasets. (**F**) The gene expression profile of the two groups of radiotherapy patients. (**G**) Time‐dependent ROC curve for OS.

The LASSO coefficients of these genes are shown in Supplementary Table S3. The risk score and survival times of LGGs patients are shown in Fig. 3A,B. A heat map of 21 genes is shown in Fig. 3C. Accordingly, patients with a risk score of − 0.183535 or lower were assigned to the RS group, which had a higher survival rate after radiotherapy. Patients with risk scores higher than − 0.183535 were classified into the RR group, which had a poorer prognosis after radiotherapy (Fig. 3D). In addition, considering the clinical difference between the RS and RR groups, we matched the two groups of patients for clinical factors, including age, sex, grade and IDH mutations status. The Kaplan–Meier curve showed that in the matched radiotherapy patients, the OS rate of the RS group was significantly higher than that of the RR group (Fig. 3E). For patients without radiotherapy before or after matching, the two groups had similar OS rates (Supplementary Fig. S2A,B). This result indicated that this signature could predict the benefit of radiotherapy, and was RT-specific. The gene expression profiles of the two groups of radiotherapy patients are shown in Fig. 3F. ROC curves showed that the prediction accuracy of the radiosensitivity signature was 0.889 (95% CI 0.818–0.960) at 3 years and 0.842 (95% CI 0.763–0.920) at 5 years (Fig. 3G).

We found that this prediction model was also applicable for the prediction of PFS in LGGs patients. The AUC values of 3- and 5-year survival were 0.791 (95% CI 0.719–0.86) and 0.842 (0.770–0.913), respectively (Supplementary Fig. S3).

### Validation of the radiosensitivity prediction signature with the CGGA datasets

**Figure 4:**
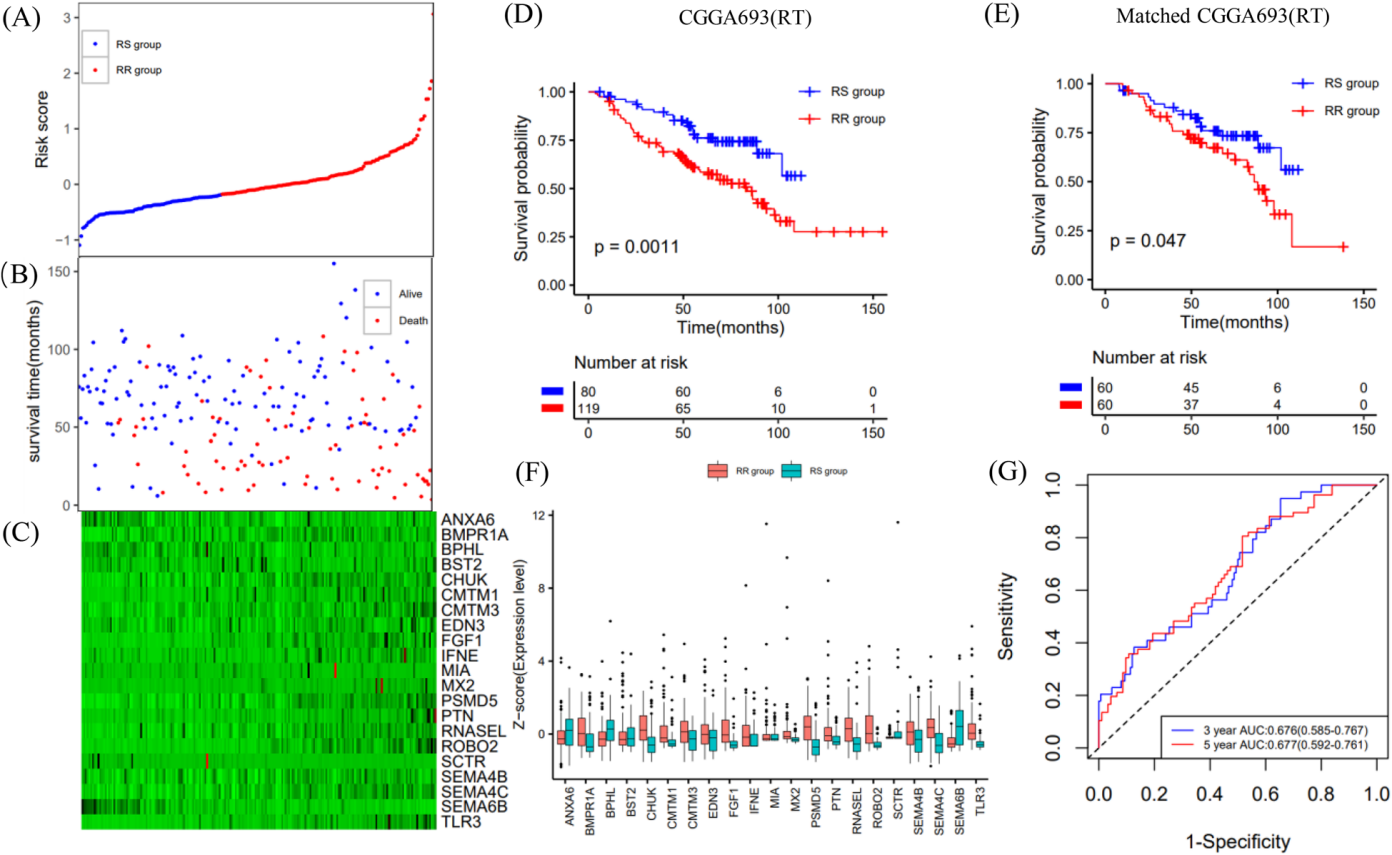
Validation of the OS prediction model based on 21 genes in the CGGA693 dataset. (**A**,**B**) The risk score, survival time of each sample. (**C**) Heatmap of 21 genes. (**D**,**E**) Survival curve of the RS group and RR group in radiotherapy patients in the unmatched and matched datasets. (**F**) The gene expression profile of the two groups of radiotherapy patients. (**G**) Time‐dependent ROC curve for OS.

**Figure 5:**
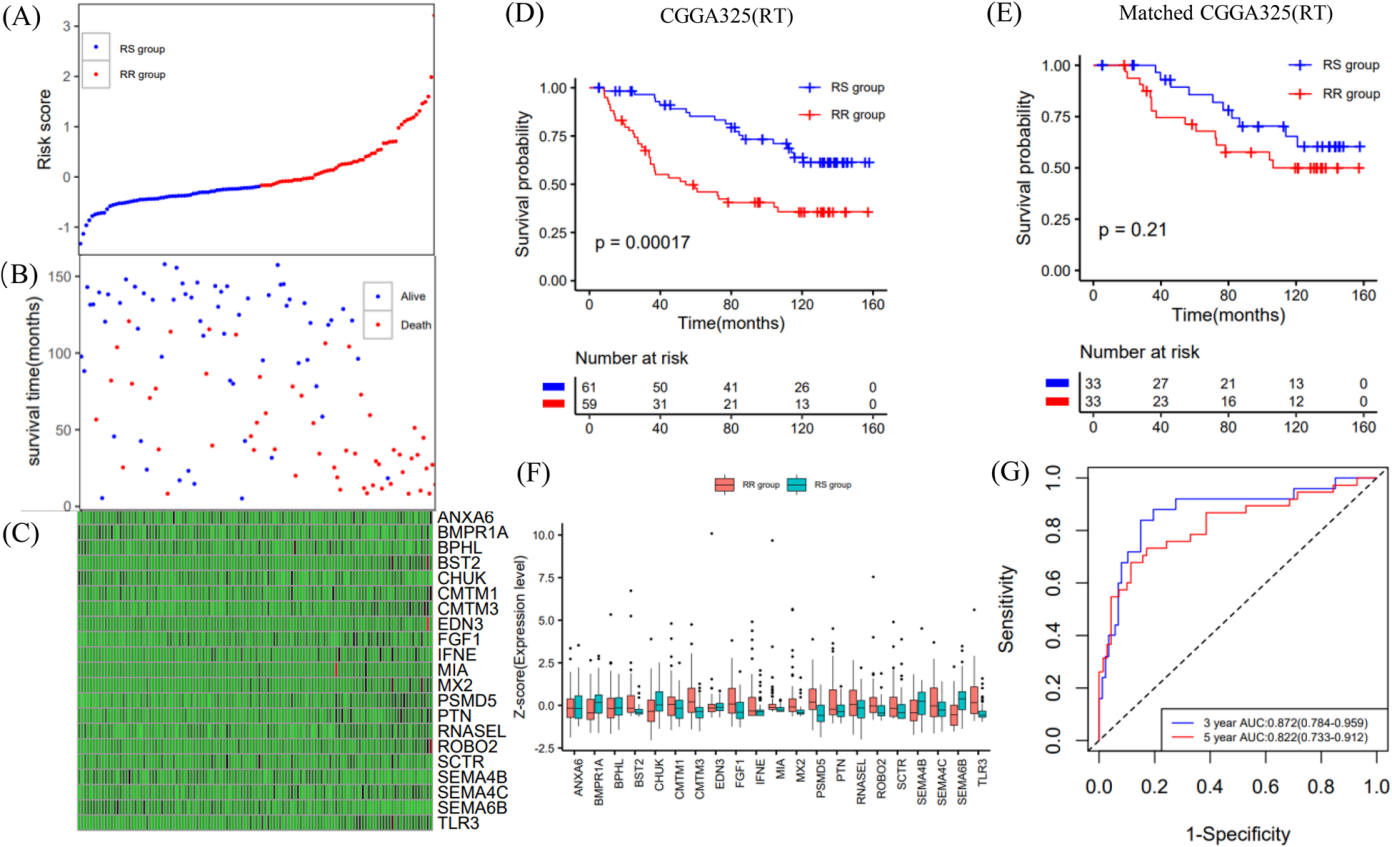
Validation of the OS prediction model based on 21 genes in the CGGA325 dataset. (**A**,**B**) The risk score, survival time of each sample. (**C**) Heatmap of 21 genes. (**D**,**E**) Survival curve of the RS group and RR group in radiotherapy patients in the unmatched and matched datasets. (**F**) The gene expression profile of the two groups of radiotherapy patients. (**G**) Time‐dependent ROC curve for OS.

Patients in the CGGA693 and CGGA325 datasets were classified into RS and RR groups using the median value (− 0.183535) established above (Figs. 4A, 5A). The survival time of each patient is shown in Figs. 4B and 5B. The expression patterns of the 21 IRGs are shown in Figs. 4C and 5C.

In the CGGA693 validation cohort, among patients receiving radiotherapy in the unmatched or matched dataset, the RS group had significantly better OS than the RR group (Fig. 4D,E). Conversely, in the matched and unmatched nonradiotherapy groups, the RS group did not show a survival benefit (Supplementary Fig. S2E,F). The gene expression profiles of the two groups of radiotherapy patients are shown in Fig. 4F. The AUC values of 3- and 5-year survival were 0.676 (95% CI 0.585–0.767) and 0.677 (95% CI 0.592–0.761), respectively (Fig. 4G).

In the CGGA325 validation cohort, the OS of patients receiving radiotherapy in the RS group was significantly prolonged compared with that in the RR group (Fig. 5D). However, perhaps due to the limited sample size, there was no significant survival difference between the two groups for the matched radiotherapy patients (Fig. 5E). In addition, in the nonradiotherapy groups, the OS rates in the RS and RR groups were similar (Supplementary Fig. S2G). Considering the small sample size, we did not match the two groups of patients without radiotherapy in the CGGA325 dataset. The gene expression profiles of the two groups of radiotherapy patients are shown in Fig. 5F. The AUC values of 3- and 5-year survival were 0.872 (95% CI 0.784–0.959) and 0.822 (95% CI 0.733–0.912), respectively (Fig. 5G).

Among all CGGA patients, patients with lower risk scores were assigned to the RS group, which had a better prognosis after radiotherapy. Conversely, in the nonradiotherapy patients, there were no significant survival differences between the two groups. The results were consistent before and after matching. The AUC values of 3- and 5-year survival were 0.782 (95% CI 0.721–0.843) and 0.757 (95% CI 0.697–0.817), respectively. (Supplementary Fig. S4).

### The associations between risk score and clinical factors

To evaluate whether this signature can be an independent OS and PFS predictor for LGGs patients receiving radiotherapy, multivariate Cox regression analyses were performed in the TCGA and CGGA datasets. The results showed that this radiosensitivity signature was an independent prognostic indicator for the TCGA-LGGs and CGGA693 datasets (Supplementary Tables S5–S7). For the CGGA325 dataset, this signature was not determined to be an independent factor by multivariate Cox regression analysis (Supplementary Table S8). However, in all CGGA patients, multivariate Cox regression analyses showed that this radiosensitivity signature was still an independent prognostic indicator (Supplementary Table S9).

### Construction of a nomogram

**Figure 6:**
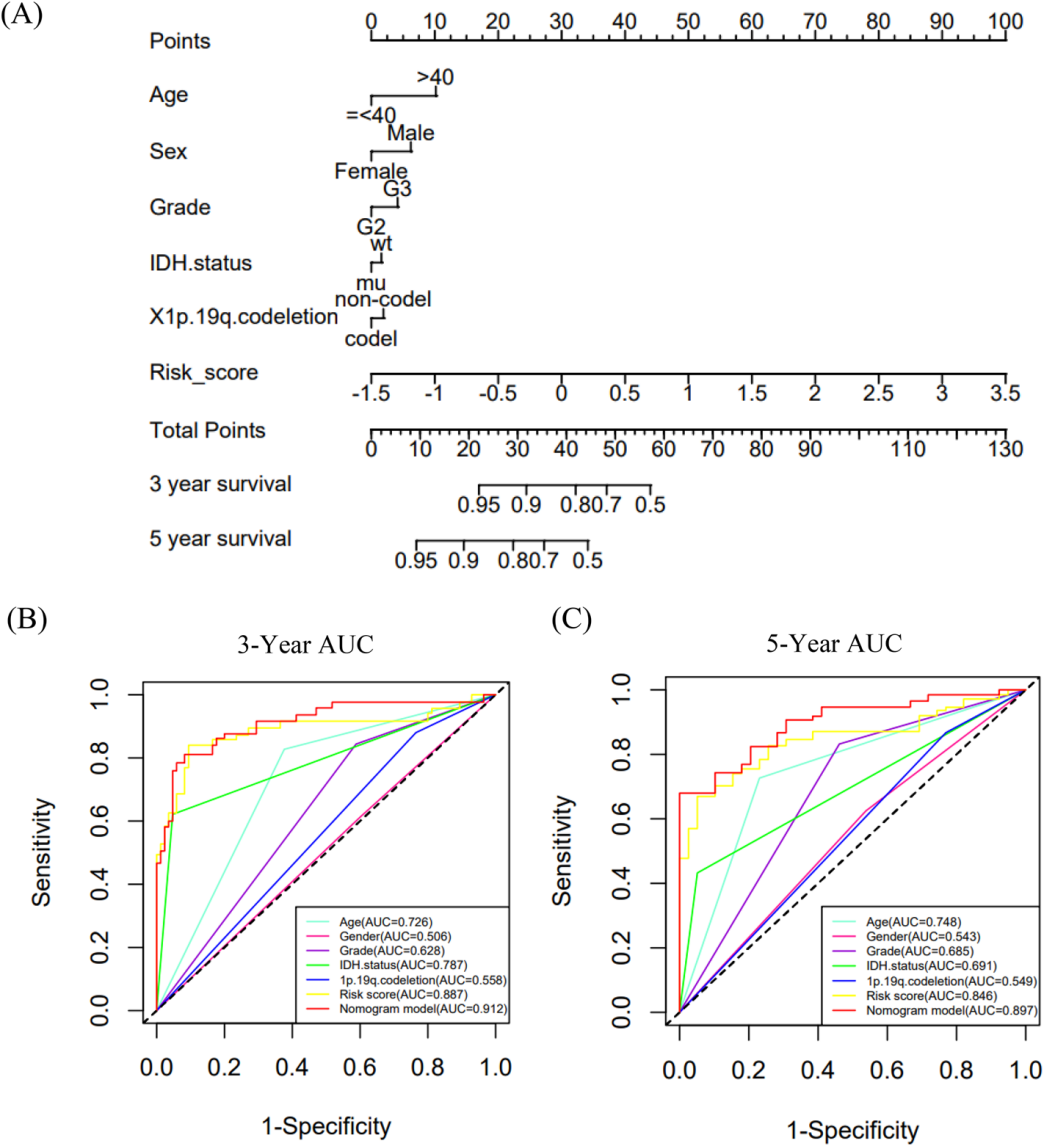
The nomogram to predict 3‐ or 5‐year OS in LGGs. (**A**) A nomogram to predict the 3- and 5-year OS of LGGs patients treated with radiotherapy in the TCGA dataset. (**B**,**C**) ROC curves comparing the prediction accuracy of each factor in the nomogram.

**Figure 7:**
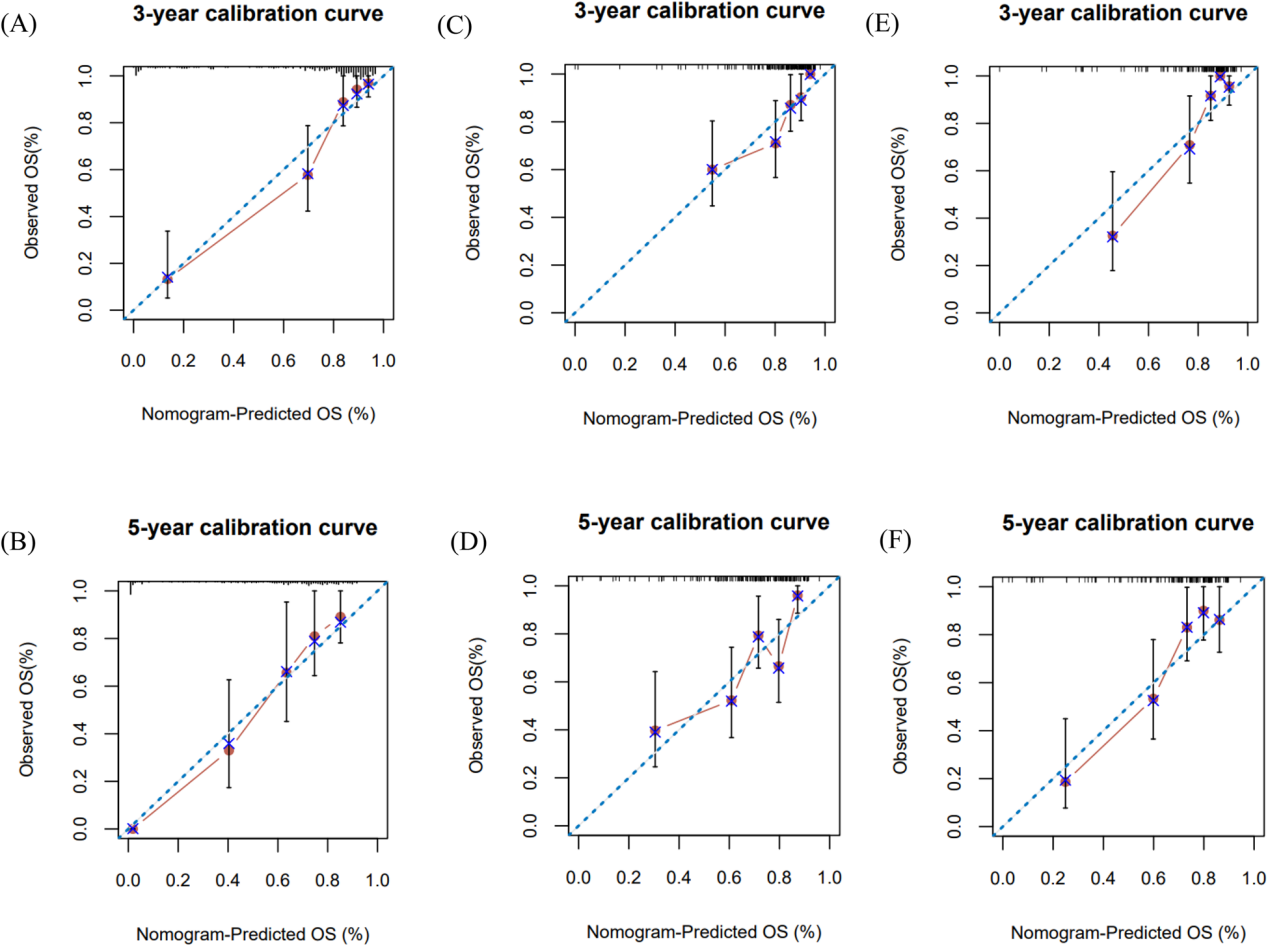
The calibration plot for validation of the nomogram. (**A**,**B**) TCGA-LGGs dataset (**C**,**D**) CGGA693 dataset (**E**,**F**) CGGA325 dataset.

We also developed a nomogram containing the risk score and clinical factors (age, sex, grade, IDH mutations and 1p/19q codeletion) in the TCGA-LGGs dataset. The nomogram was used to predict 3-year and 5-year OS rates (Fig. 6A). The AUC values of the nomogram were 0.912 and 0.897 for the 3-year and 5-year OS rates (Fig. 6B,C), respectively. The AUC curve results of the CGGA693 and CGGA325 datasets are shown in Supplementary Fig. S5. The risk score showed a better predictive value than other clinical factors, and the AUC values of the 3-year and 5-year OS rates of the nomogram were larger than those of a single predictor. The calibration curves of 3-year and 5-year OS indicated that the nomogram had good prediction accuracy for both the TCGA and CGGA datasets (Fig. 7).

### Comparison with previously published gene signatures

We compared two existing gene signatures for predicting radiotherapy response, an Rscore^25^ and the radiation sensitivity index (RSI)^26^, in the TCGA and CGGA datasets. For the RSI, the 25th percentile of RSI in patients receiving radiotherapy was used as the cutoff value for dividing patients into RS and RR groups, as in previous studies^22,27^. RSI did not show significant results in predicting radiotherapy benefits in either the TCGA or CGGA datasets (Supplementary Fig. S6). The Rscore could distinguish which patients would benefit from radiotherapy and predict the OS of patients receiving radiotherapy. However, the AUC of the Rscore was less than the immune-related signature (5 AUCs: TCGA [0.729 vs. 0.842], CGGA639 [0.606 vs. 0.677], CGGA325 [0.695 vs. 0.822], CGGA [0.663 vs. 0.757]) (Supplementary Fig. S7, Supplementary Table S10).

### Immune cell subtypes of the radiosensitive and radioresistant groups

**Figure 8:**
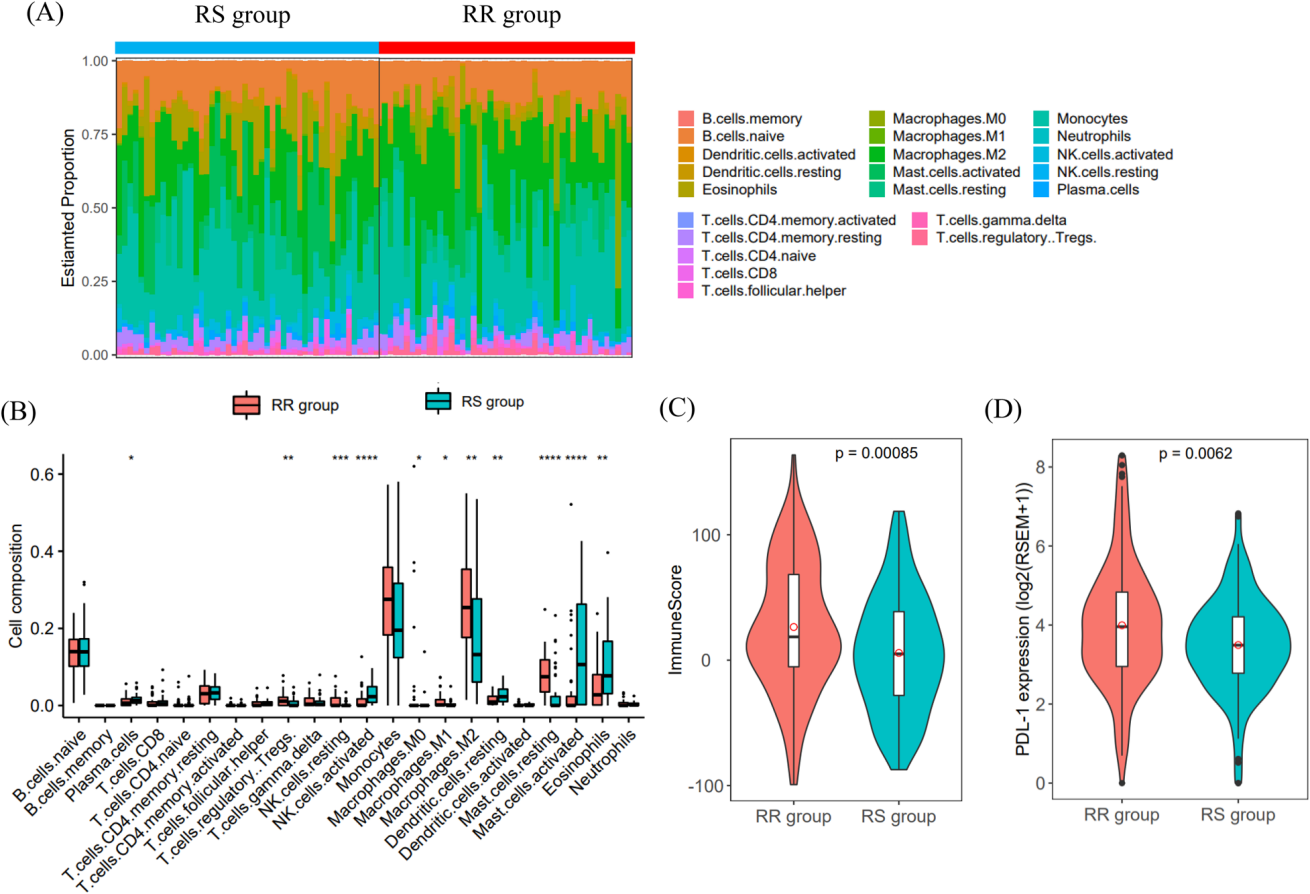
Tumor-infiltrating immune cell analysis. (**A**) The percentages of 22 immune cells in the RR and RS groups of radiotherapy patients in the TCGA-LGGs dataset. (**B**) Comparison of immune cell proportions between the RS and RR groups (RS, 48 samples; RR, 46 samples). (**C**,**D**) Comparison of immune score and PD-L1 expression between the RS and RR groups.

To understand the relationship between the radiosensitivity signature identified based on the immune gene list and the tumor microenvironment, the CIBERSORT algorithm was used to compute the proportions of 22 immune cell subtypes in the two groups (Fig. 8A). The infiltration levels of plasma cells, activated NK cells, resting dendritic cells, activated mast cells, and eosinophils were significantly higher in the RS group. Conversely, in the RR group, the infiltration levels of macrophages (M0, M2), Treg cells, resting mast cells, and resting NK cells were significantly higher (Fig. 8B). Among patients receiving radiotherapy, the immune score of the RR group was significantly higher than that of the RS group and PD-L1 expression was upregulated in the RR group. These results indicated that the patients in the RR group showed higher immunogenicity (Fig. 8C,D). Similarly, in the CGGA datasets, the immune score and PD-L1 expression level of the RR group were higher than those of the RS group. Although the infiltration level of these 10 immune cells was not significantly different between the RS and RR groups, similar trends were evident (Supplementary Figs. S8, S9).

### GSEA

GSEA showed 4 significant KEGG pathways related to the risk score, including the GPCR ligand binding pathway, class A/1 rhodopsin-like receptor pathway, peptide ligand binding receptor pathway, and GPCR signaling pathway (Supplementary Fig. S10). These signaling pathways are mainly related to the development of tumors or metastasis.

## Discussion

With the introduction of advanced technology, radiotherapy methods have significantly improved, increasing the accuracy of radiotherapy. Despite these advances, radiotherapy resistance remains an important issue, and the response of cancer patients to radiotherapy varies greatly^28^. Predictive biomarkers provide a method to stratify patients receiving a specific treatment and identify those who respond well to that treatment. In the era of precision medicine, gene expression profiling has been a useful tool to explore the radiosensitivity of individual patients^27,29,30^. Traditionally, research on radiosensitivity has mainly focused on tumor cells, while ignoring the effect of the tumor microenvironment composed of stromal and immune cells^31^. Recent studies have indicated that the immune system has an important influence on cancer patients' response to treatment and long-term prognosis^32,33^.

In this research, we used the LASSO Cox regression model to establish an immune radiosensitivity signature and constructed a nomogram to estimate the OS rate of LGGs patients receiving radiotherapy. The TCGA-LGGs dataset was used to confirm that 21 IRGs (ANXA6, BMPR1A, BPHL, BST2, CHUK, CMTM1, CMTM3, EDN3, FGF1, IFNE, MIA, MX2, PSMD5, PTN, RNASEL, ROBO2, SCTR, SEMA4B, SEMA4C, SEMA6B, and TLB3) could identify patients who were most likely to benefit from radiotherapy. The CGGA datasets were used as independent validation sets. The results indicated that this signature could stratify patients according to the prediction of the benefit of radiotherapy.

With the TCGA-LGGs dataset, 21 IRGs were identified using the LASSO Cox regression model. The risk score of each patient was computed based on the mRNA expression of 21 genes and LASSO regression coefficients. We showed that among patients who received radiotherapy, patients with lower risk scores had a higher OS rate than patients with higher risk scores. In contrast, there was no significant survival difference between the two groups that did not receive radiotherapy. We showed that the signature was RT-specific and only predicts outcome in RT-treated patients. The radiosensitivity signature was also applicable when PFS was used as the research endpoint. However, there was no PFS information in the CGGA datasets, and this result could not be validated with external data. Meanwhile, we performed an exact 1:1 match between the high- and the low-risk groups, which ensured the balance of the cohort and allowed for more rigorous validation. The conclusion was consistent with the above. Therefore, patients with lower risk scores were assigned to the RS group, and other patients were assigned to the RR group. Furthermore, this signature was still an independent prognostic indicator in the multivariate Cox regression analysis of the TCGA-LGGs and CGGA693 datasets. However, in the CGGA325 dataset, the risk score was not an independent factor. This finding may be due to the limited number of patients in the CGGA325 dataset and still needs further validation. Therefore, we combined the two CGGA datasets to increase the sample size of the validation dataset. The results of all CGGA patients indicated that this immune-related gene signature could identify patients most likely to benefit from radiotherapy and had high accuracy in predicting survival after radiotherapy.

To clarify the influence of TME function on the effect of radiotherapy in patients with LGGs, we calculated the profiles of immune cell composition in the RS and RR groups. The infiltration levels of 10 types of immune cells in the RS group and RR group were significantly different. As our results showed, the activated NK cell infiltration level was significantly higher in the RS group. In contrast, the infiltration levels of macrophages (M0, M2), Treg cells, and resting NK cells in the RR group were significantly increased. In addition, the immune score and PD-L1 expression level of the RR group were significantly higher than those of the RS group. These results indicated that the patients in the RR group showed higher immunogenicity. Radiation exerts immunostimulatory activity by increasing the cytotoxicity of NK cells, increasing tumor infiltration and cytokine production, increasing the accumulation of M1 macrophages, and reducing the level of Treg lymphocytes^10,34^. NK cells are the body's first line of defense against tumor cells. Low-dose radiation can adjust the sensitivity of NK cells to tumor cells, thereby increasing tumor lethality^35^. In addition, Treg cells are associated with radioresistance. Treg cells are an important cell type of the immune system and are related to the immunosuppressive phenotype of cancer patients. Several studies on the radiosensitivity of Treg cells have shown that they are more radioresistant than other T or B lymphocyte subpopulations^36,37^. M2 macrophages, also called activated macrophages, aid in tissue repair, matrix remodeling and angiogenesis and can suppress the immune system^38^. Studies have shown that M2 cells are related to chemoresistance^39^ and radioresistance^40^. Cancer treatment is not a static process; it is related to the surrounding environment that supports the tumor. Therefore, the success of effective antitumor therapy depends on the integration of targeted antitumor strategies and antitumor environmental strategies and the use of possible synergy between them^10^.

Recently, a three-lncRNA signature^41^ and a five-microRNA signature^42^ were used as independent biomarkers for predicting LGGs patient outcomes after radiotherapy. Patients who had higher risk scores tended to have shorter survival times after radiotherapy treatment. In this study, we used the TCGA-LGGs dataset to generate a 21-gene signature that can predict the sensitivity of LGGs patients to radiotherapy. However, the survival difference between the high- and low-risk score groups might not indicate radiosensitivity. It is easy to overlook that the OS of these two groups of patients might also differ among nonradiotherapy patients. Therefore, it is necessary to compare the survival rate between high- and low-risk score patients who did not receive radiotherapy^43^. This immune-related gene signature was designed to predict the radiosensitivity of LGGs patients because it was developed in all radiotherapy patients and also validated in patients who did not receive radiotherapy.

In addition, we compared two existing gene signatures for predicting the OS of LGGs patients after radiotherapy, an Rscore and the RSI, in the TCGA, and CGGA datasets. The RSI did not show significant results in predicting radiotherapy benefits in either the TCGA or CGGA datasets. The Rscore could distinguish which patients would benefit from radiotherapy and predict the OS of patients receiving radiotherapy. However, the AUC of the Rscore was less than that of the immune-related signature. Thus, this immune-related gene signature could identify patients most likely to benefit from radiotherapy and had higher accuracy in predicting survival after radiotherapy. If these results are confirmed in future clinical trials, this gene signature could be used to select patients who are sensitive to radiotherapy or to identify patients who may have a poor prognosis after radiotherapy. Thus, it could guide clinicians to adjust treatment plans.

Like other studies, ours inevitably had some limitations. First, our research datasets come from public datasets, and further studies containing more LGGs patients are needed. Second, our study used retrospective cohorts, and this signature was not validated in a prospective cohort. Finally, more experiments should be performed to study the function of the genes included in the signature to clarify the association between immune infiltrates and radiotherapy.

## Conclusion

In general, our research led to the successful creation of an immune-related radiosensitivity gene signature that can effectively identify LGGs patients who are most likely to benefit from radiotherapy. Furthermore, we constructed a nomogram based on the gene score and clinical variables that could be a useful tool for estimating the survival of LGGs patients after radiotherapy. This signature is a powerful supplement to the prognostic factors of LGGs patients with radiotherapy and may provide an opportunity to incorporate individual tumor biology into clinical decision making in radiation oncology.

## Supplementary Information

Supplementary Figures.

Supplementary Tables.

## Data Availability

All data used to support our findings are available from The Cancer Genome Atlas database (http://cancergenomec.nih.gov/), the Chinese Glioma Genome Atlas (CGGA) (http://www.cgga.org.cn), and the immunology database and analysis portal (ImmPort) (https://www.immport.org/).
