# Supplementary material for: Development and validation of an immune-related gene signature for predicting the radiosensitivity of lower-grade gliomas
Source: Sci Rep. 2022 Apr 23;12:6698. doi: 10.1038/s41598-022-10601-5 (PMC9035187; doi:10.1038/s41598-022-10601-5)
Supplement: Supplementary file 1 — Supplementary Figures. [file 41598_2022_10601_MOESM1_ESM.doc]

**Supplementary figures**

**
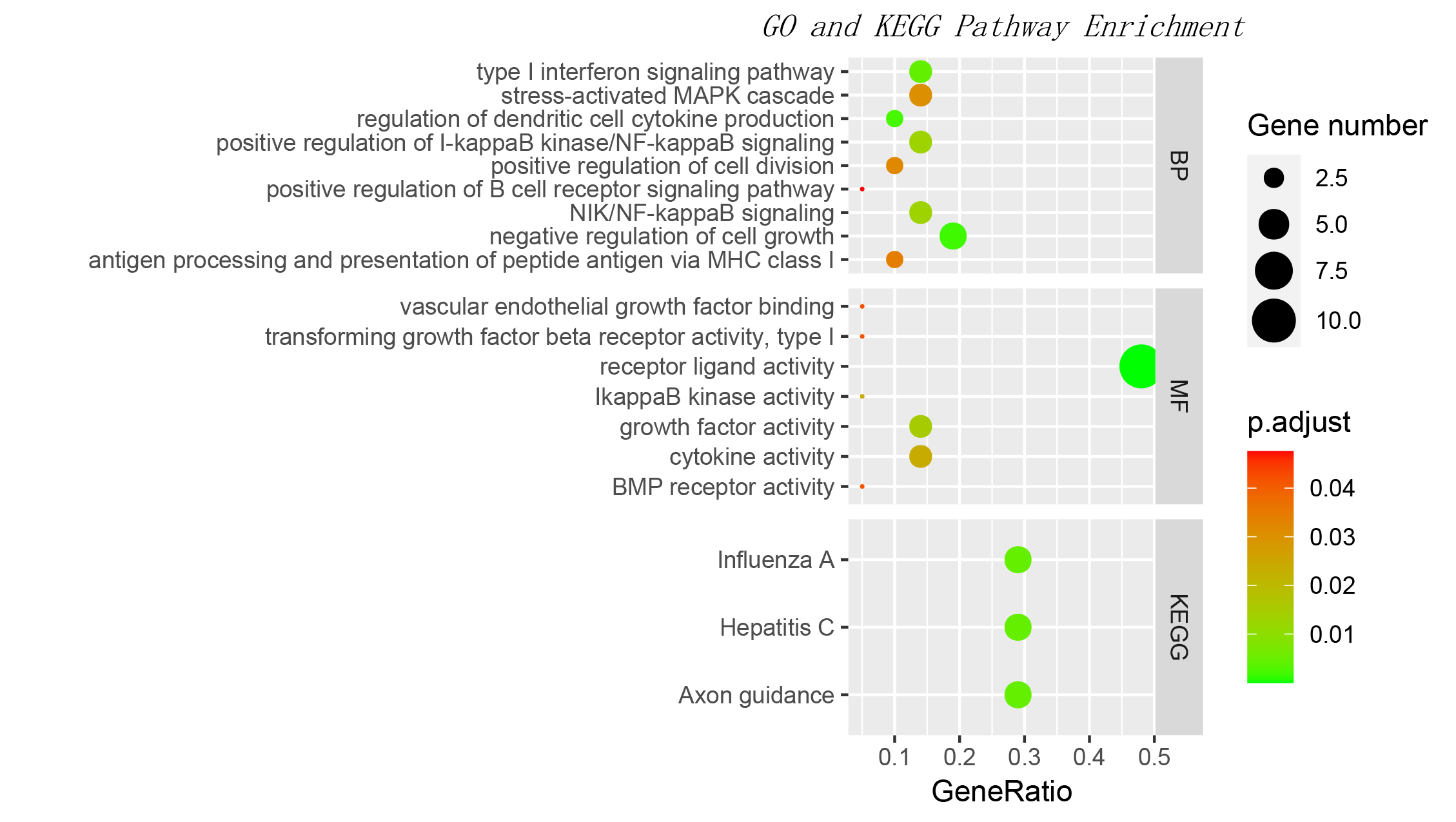
**

**Figure S1** Gene functional annotation of 21 immune-related genes. KEGG, Kyoto Encyclopedia of Genes and Genomes; BP, biological process; MF, molecular function.


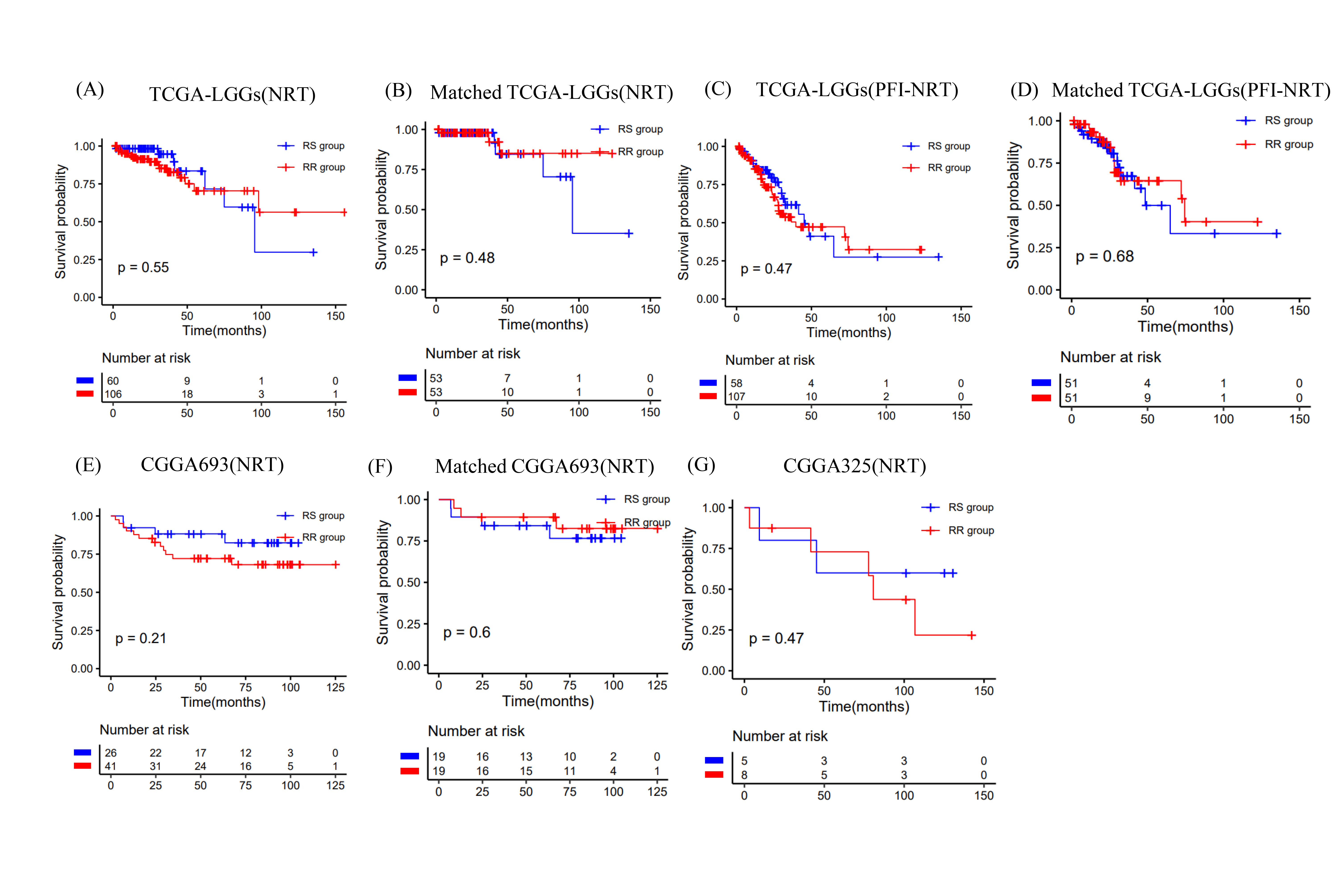


**Figure S2** Kaplan-Meier survival analysis of OS/PFS in RS group and RR group in the unmatched and matched nonradiotherapy patients. (A) unmatched TCGA-LGGs dataset. (B) matched TCGA-LGGs dataset. (C) unmatched TCGA-LGGs dataset (PFS). (D) matched TCGA-LGGs dataset (PFS). (E) unmatched CGGA693 dataset. (F) matched CGGA693 dataset. (G) unmatched CGGA325 dataset.

**
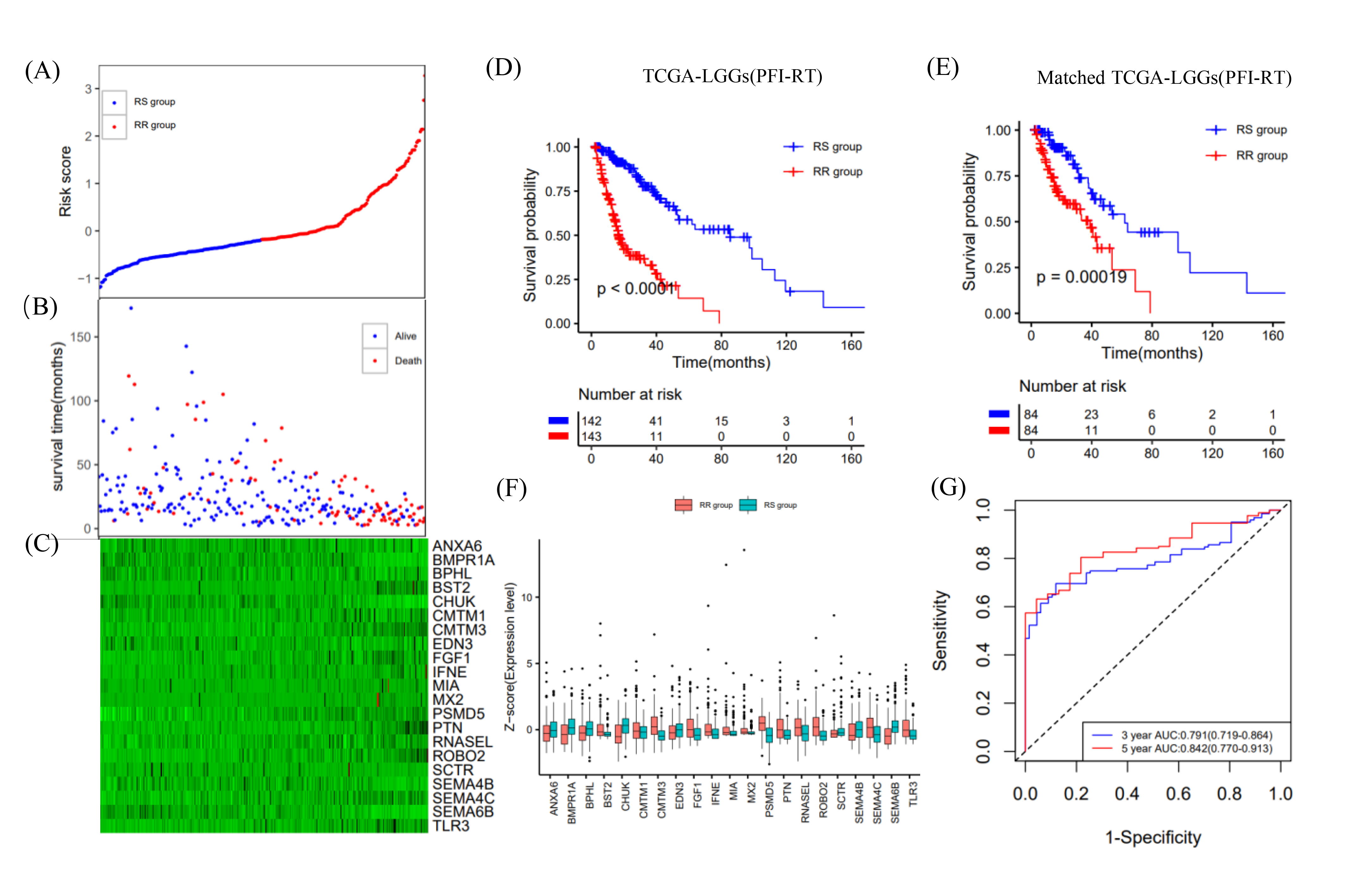
**

**Figure S3** Construction of the PFS prediction model based on 21 genes in the TCGA dataset. (A, B) The risk score, survival time of each sample. (C) Heatmap of 21 genes. (D, E) Kaplan‐Meier survival analysis of the RS group and RR group in radiotherapy patients in the unmatched and matched datasets. (F) The gene expression profile of the two groups of radiotherapy patients. (G) Time‐dependent ROC curve for PFS.


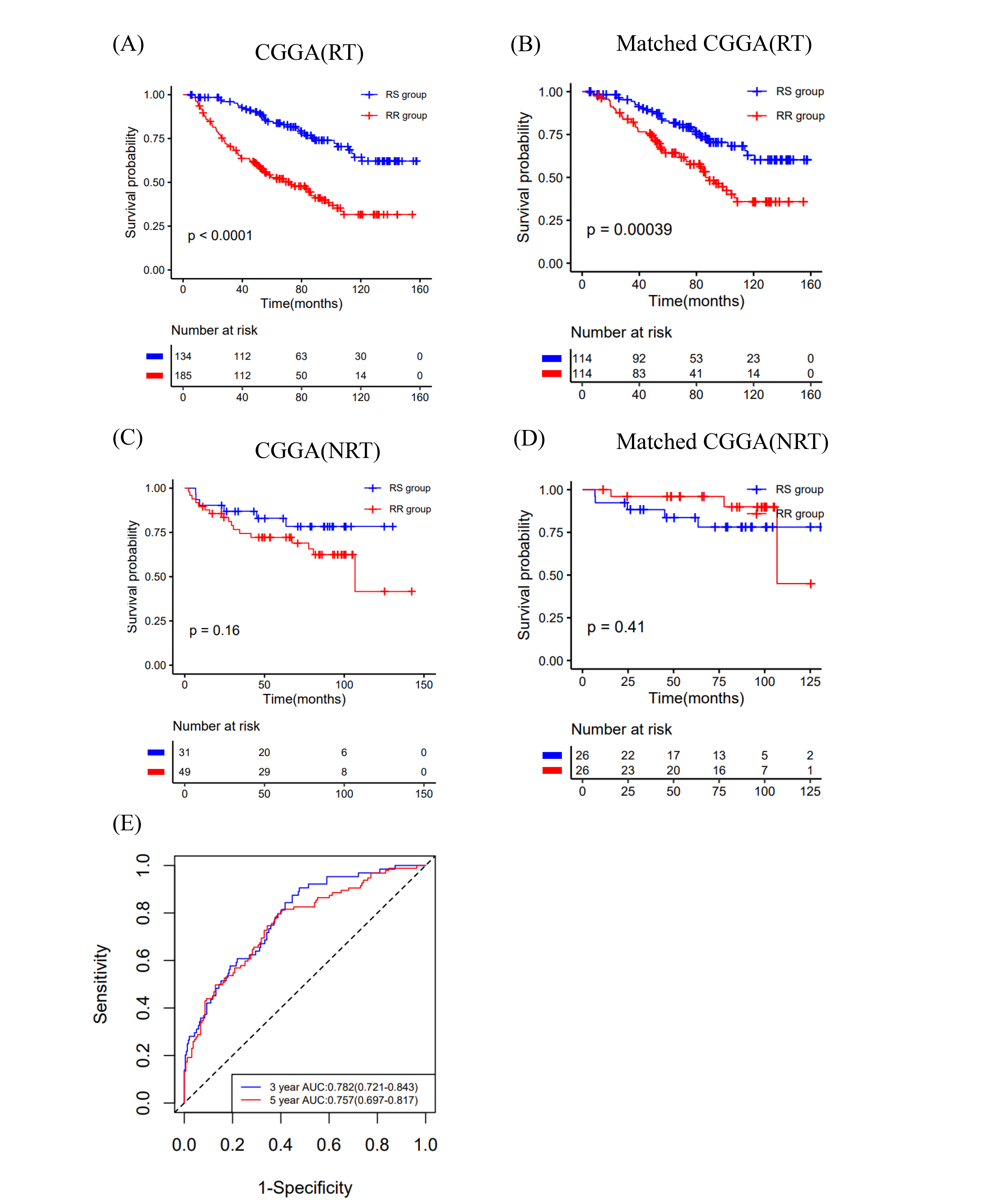
**Figure S4** Kaplan-Meier survival analysis of OS in RS group and RR group in the all CGGA patients. (A) unmatched radiotherapy patients. (B) matched radiotherapy patients. (C) unmatched nonradiotherapy patients. (D) matched nonradiotherapy patients. (E) The 3‐year and 5-year ROC curves of CGGA patients receiving radiotherapy.


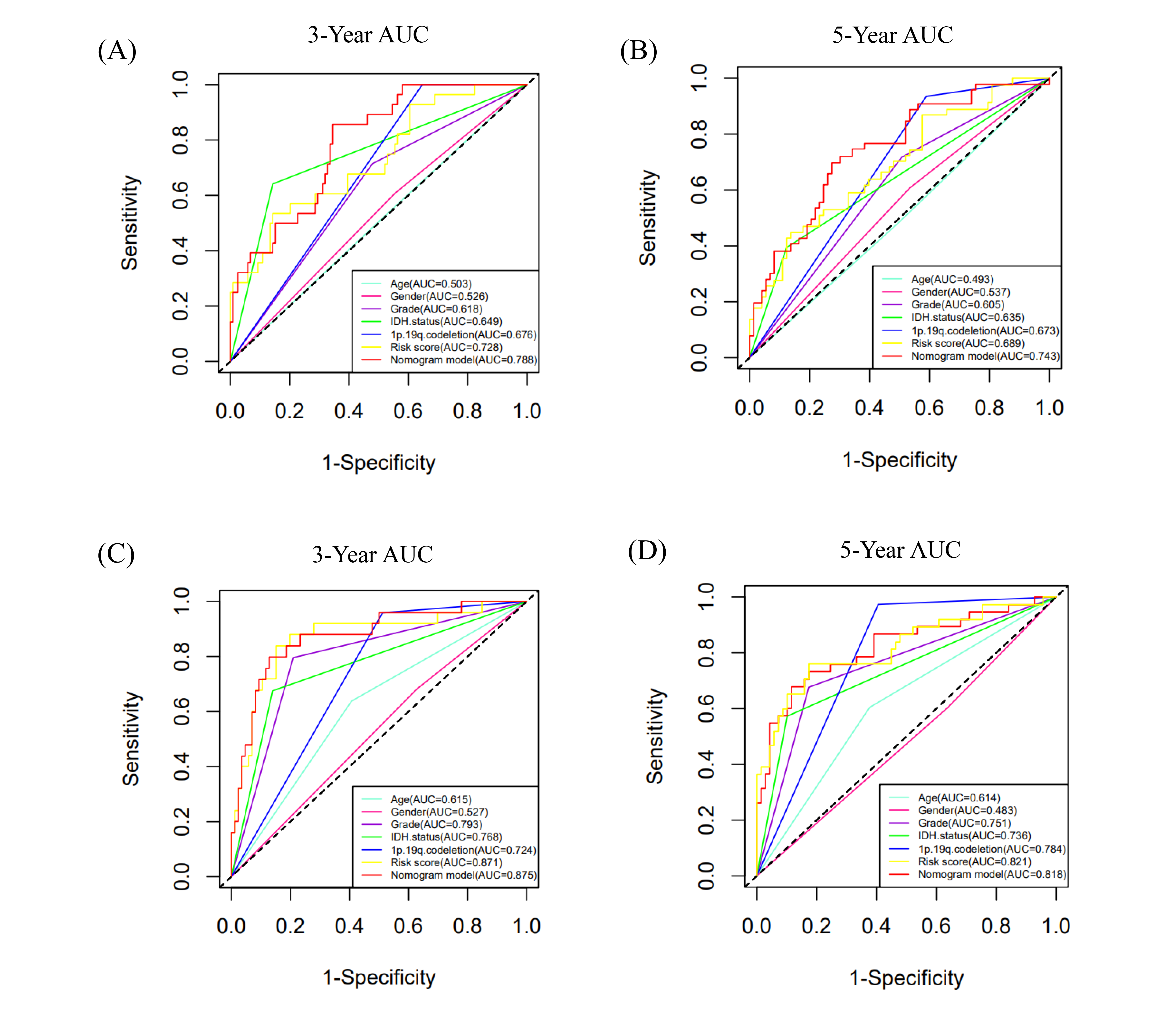
**Figure S5** (A, B) The 3‐year and 5-year ROC curves of the CGGA693 dataset. (C, D) The 3‐year and 5-year ROC curves of the CGGA325 dataset.

**
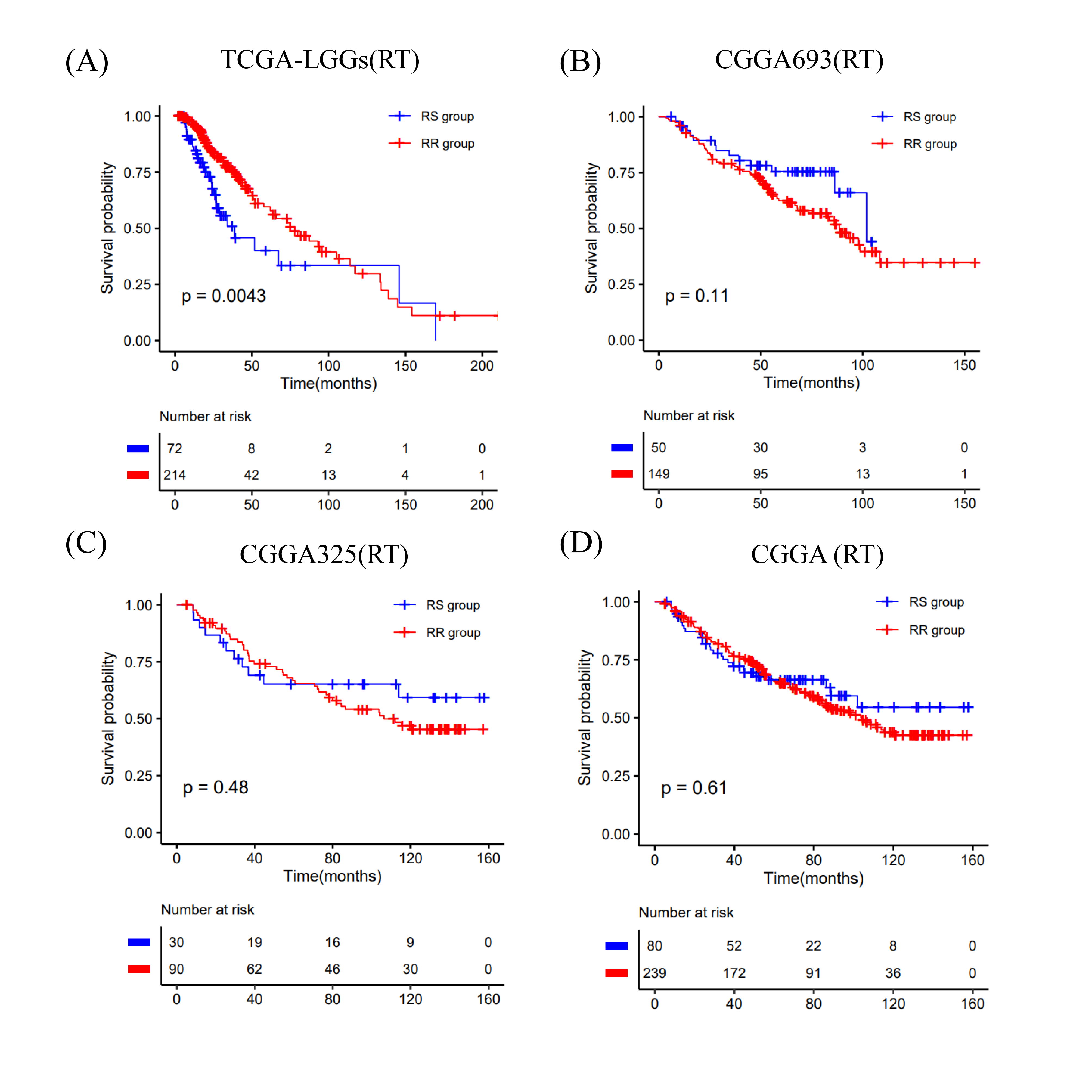
**

**Figure S6** Overall survival stratified by RSI. The survival curves comparison of RS and RR groups in the (A) TCGA dataset (B) CGGA693 dataset (C) CGGA325 dataset (D) CGGA dataset. RT, radiotherapy; RS, radiosensitive; RR, radioresistant.


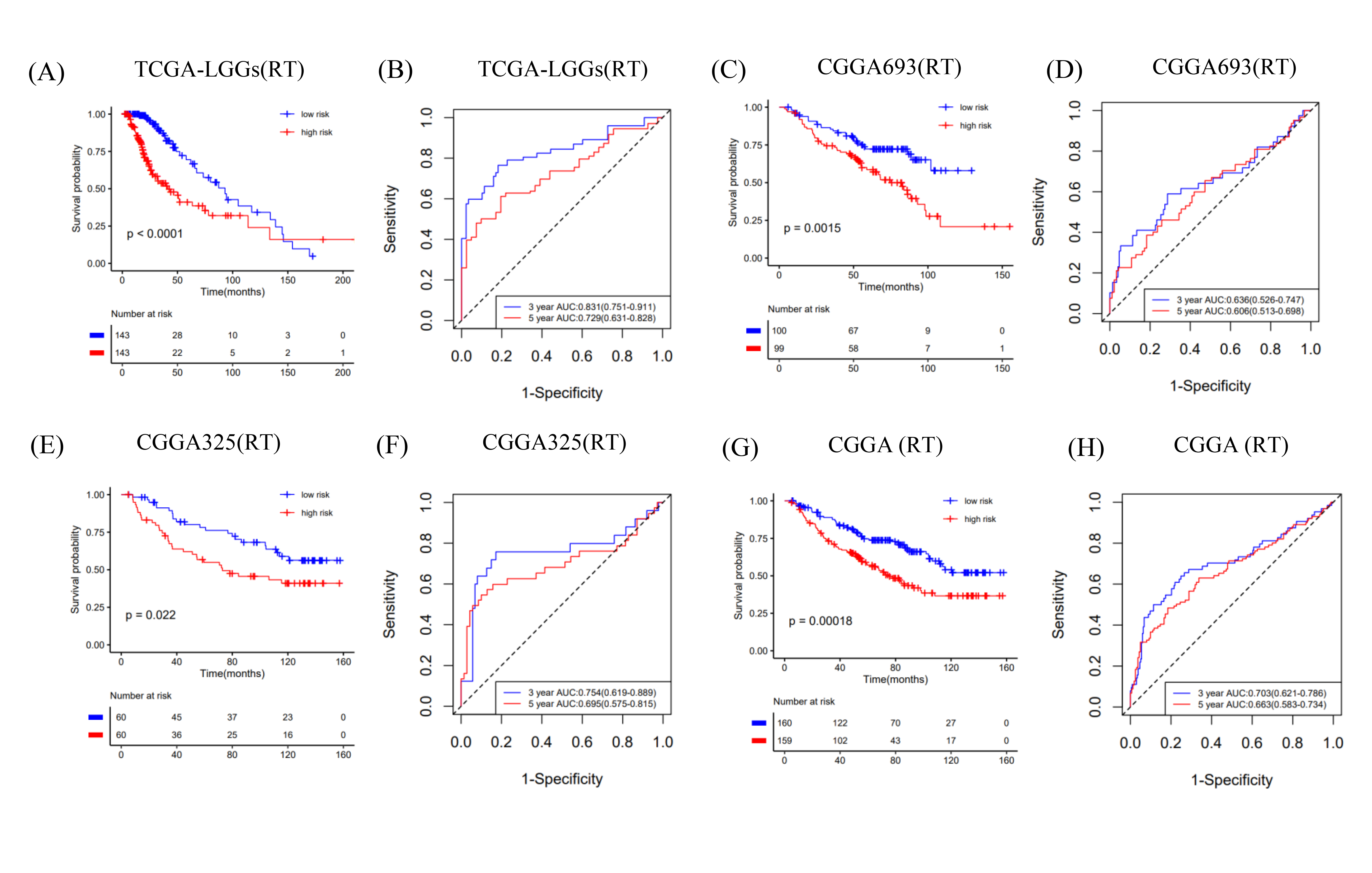
**Figure S7** Overall survival stratified by the Rscore in the TCGA and CGGA datasets. (A, C, E, G) The survival curves comparison of RS and RR groups. (B, D, F, H) The 3‐year and 5-year ROC curves of radiotherapy patients.


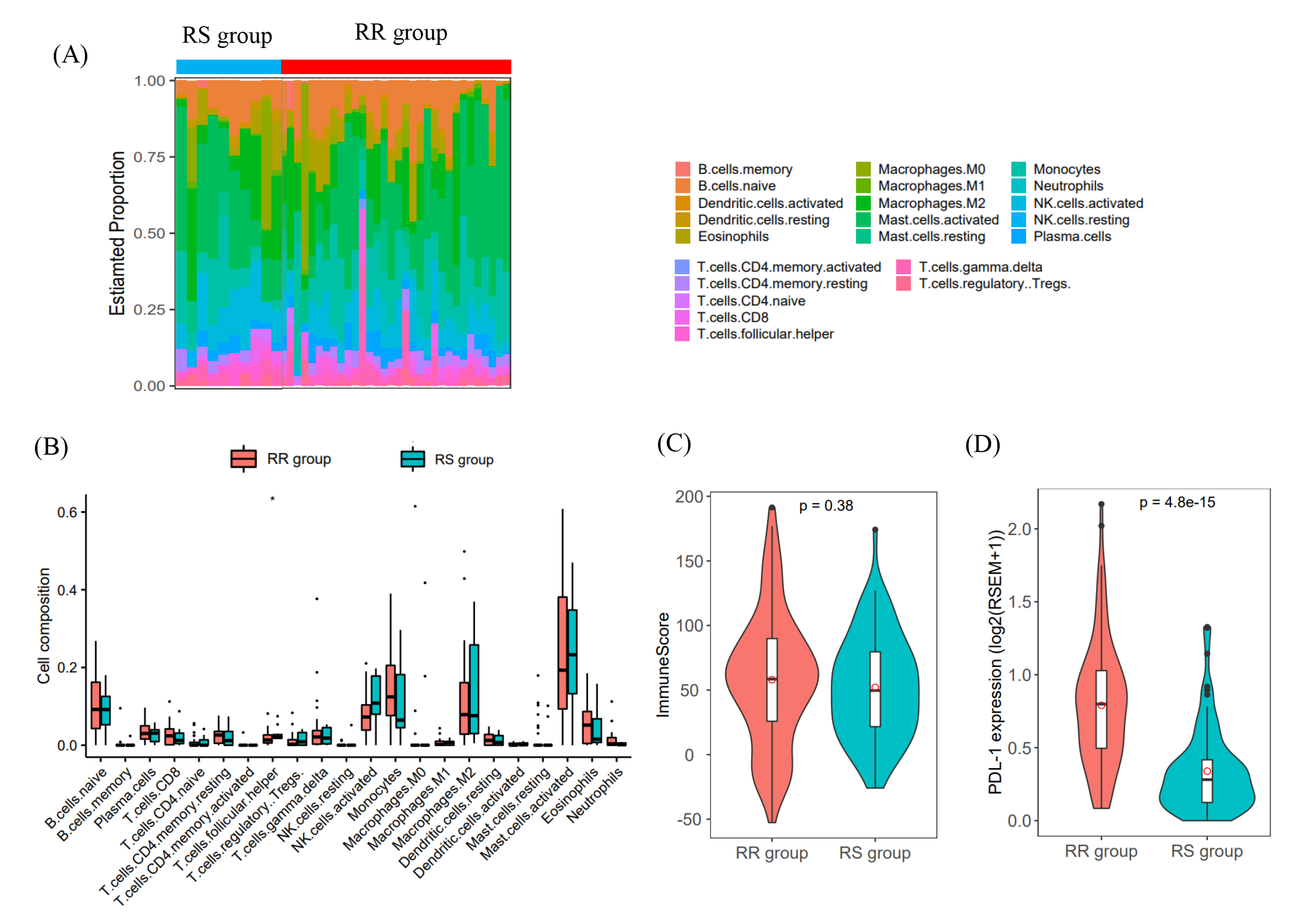
**Figure S8** (A) The percentage of 22 infiltrative immune cells in the RR and RS groups of radiotherapy patients in the CGGA693 dataset. (B) Comparison of immune cell proportions between the RS and RR groups (RS, 10 samples; RR, 32 samples). (C, D) Comparison of immune score and PD-L1 expression between the RS and RR groups.


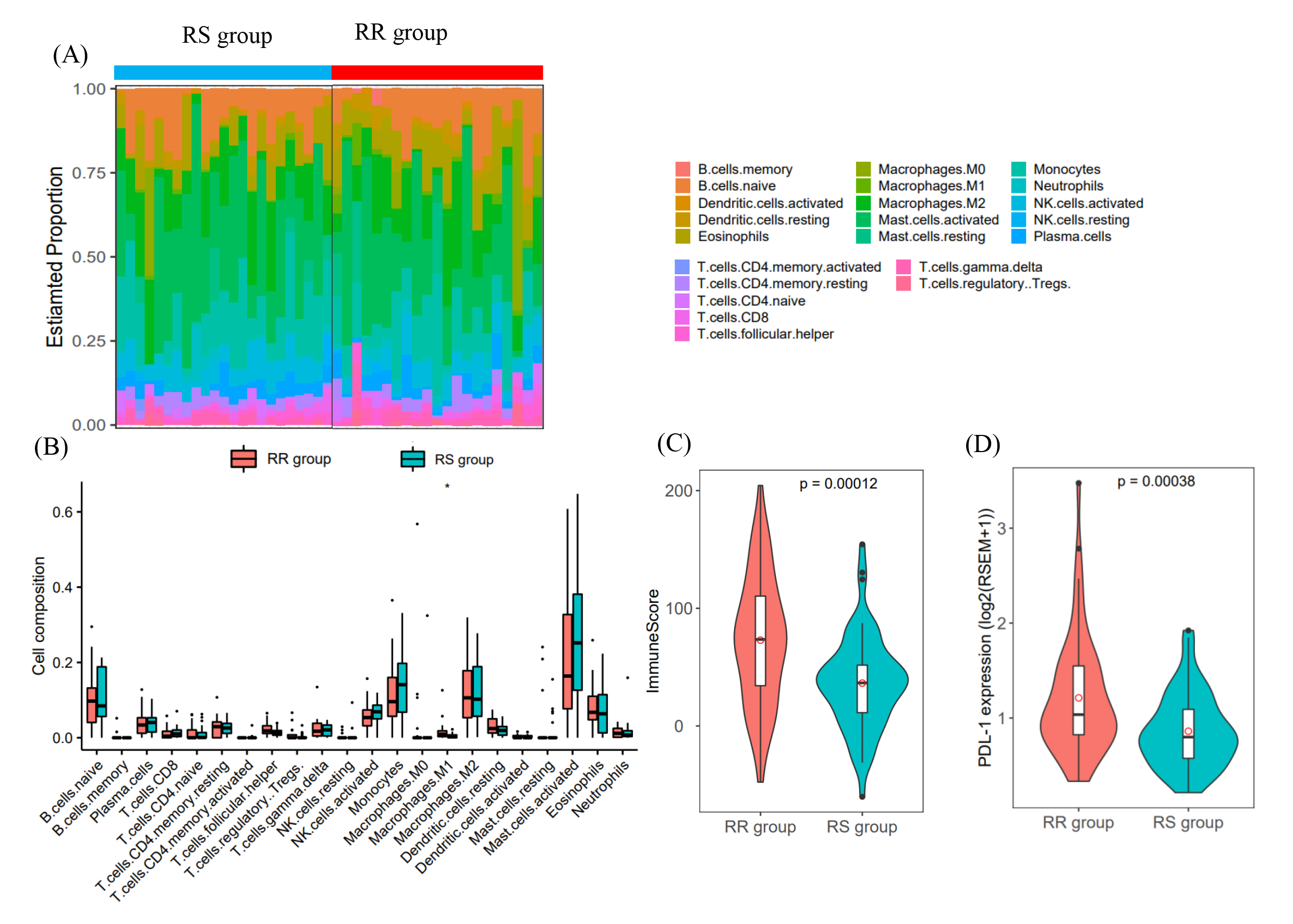
**Figure S9** (A) The percentage of 22 infiltrative immune cells in the RR and RS groups of radiotherapy patients in the CGGA325 dataset (B) Comparison of immune cell proportions between the RS and RR groups (RS, 23 samples; RR, 21 samples). (C, D) Comparison of immune score and PD-L1 expression between the RS and RR groups.

.


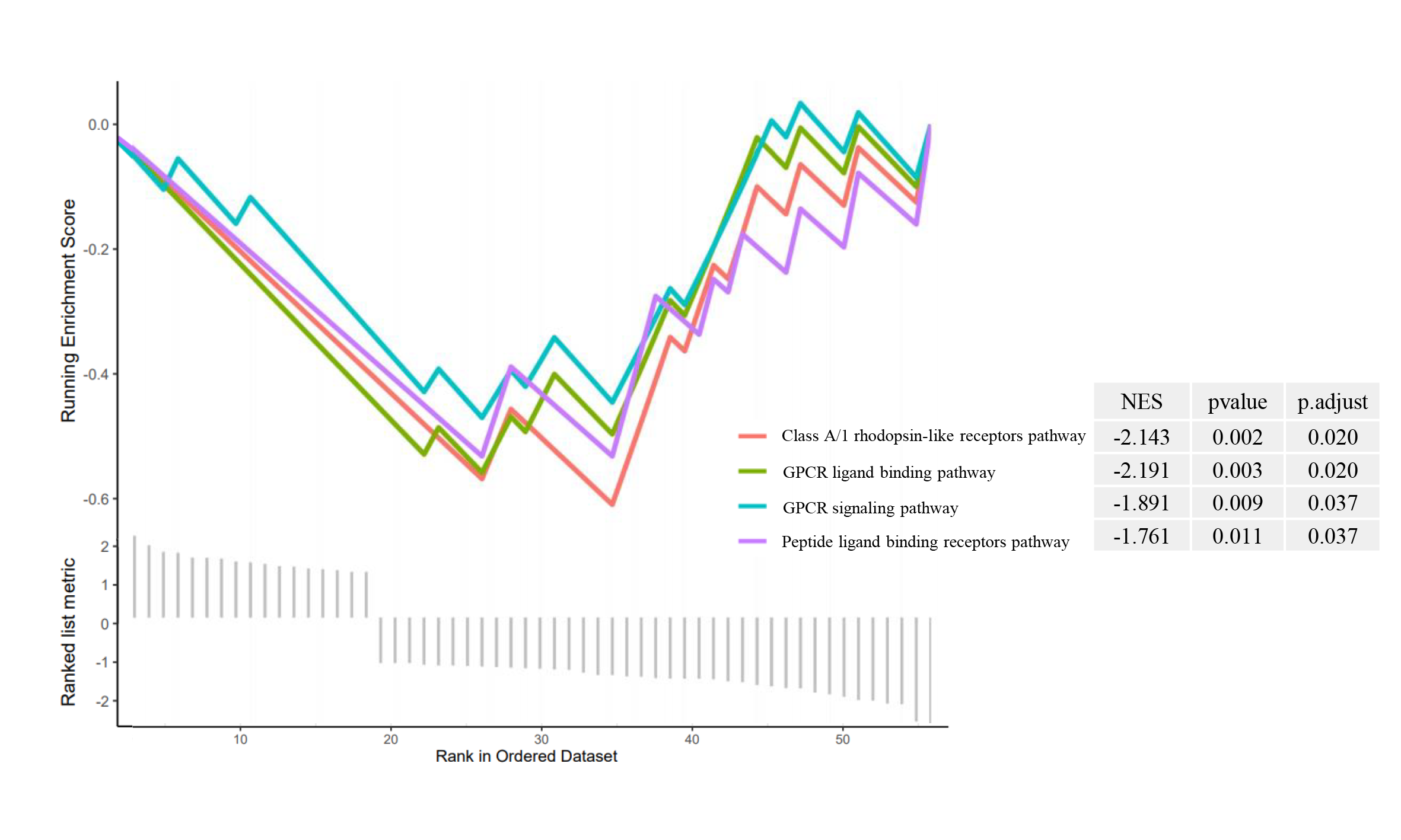


**Figure S10** Gene set enrichment analysis.
